# Supplementary figures and images for: Transcriptome dynamics along axolotl regenerative development are consistent with an extensive reduction in gene expression heterogeneity in dedifferentiated cells
Source: PeerJ. 2017 Nov 6;5:e4004. doi: 10.7717/peerj.4004 (PMC5678507; doi:10.7717/peerj.4004)

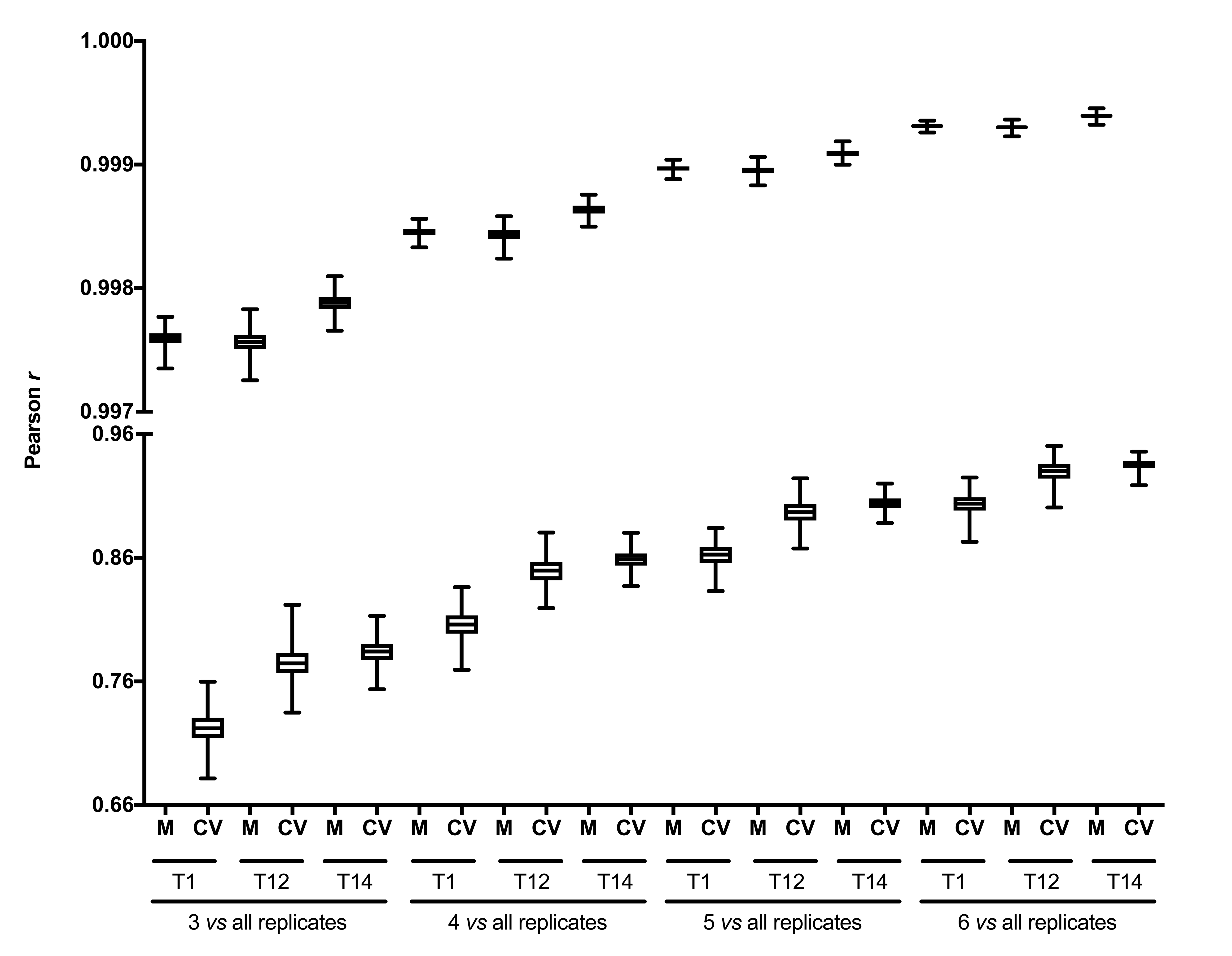

Supplement: Figure S1 — Transcriptomic data originally produced by Voss and coworkers in 2015 was retrieved from Gene Expression Omnibus database (GSE67118) (Barrett et al., 2013; Voss et al., 2015; Voss, Woodcock & Zambrano, 2015). For timepoints T1, T2, and T14, transcript abundance mean and CV were calculated for each probeset using all biological replicates and 3, 4, 5, or 6 randomly chosen biological replicates. Low-replication simulations were repeated 1,000 times. The similarity for transcript abundance measures for all probesets in each timepoint calculated using all and 3, 4, 5, or 6 biological replicates was quantified using Pearson r. Boxes represent 25th and 75th percentiles (Q1 and Q3), lines within boxes represent medians, and whiskers represent minimum and maximum. M: mean, CV: coefficient of variation. [file peerj-05-4004-s001.png]

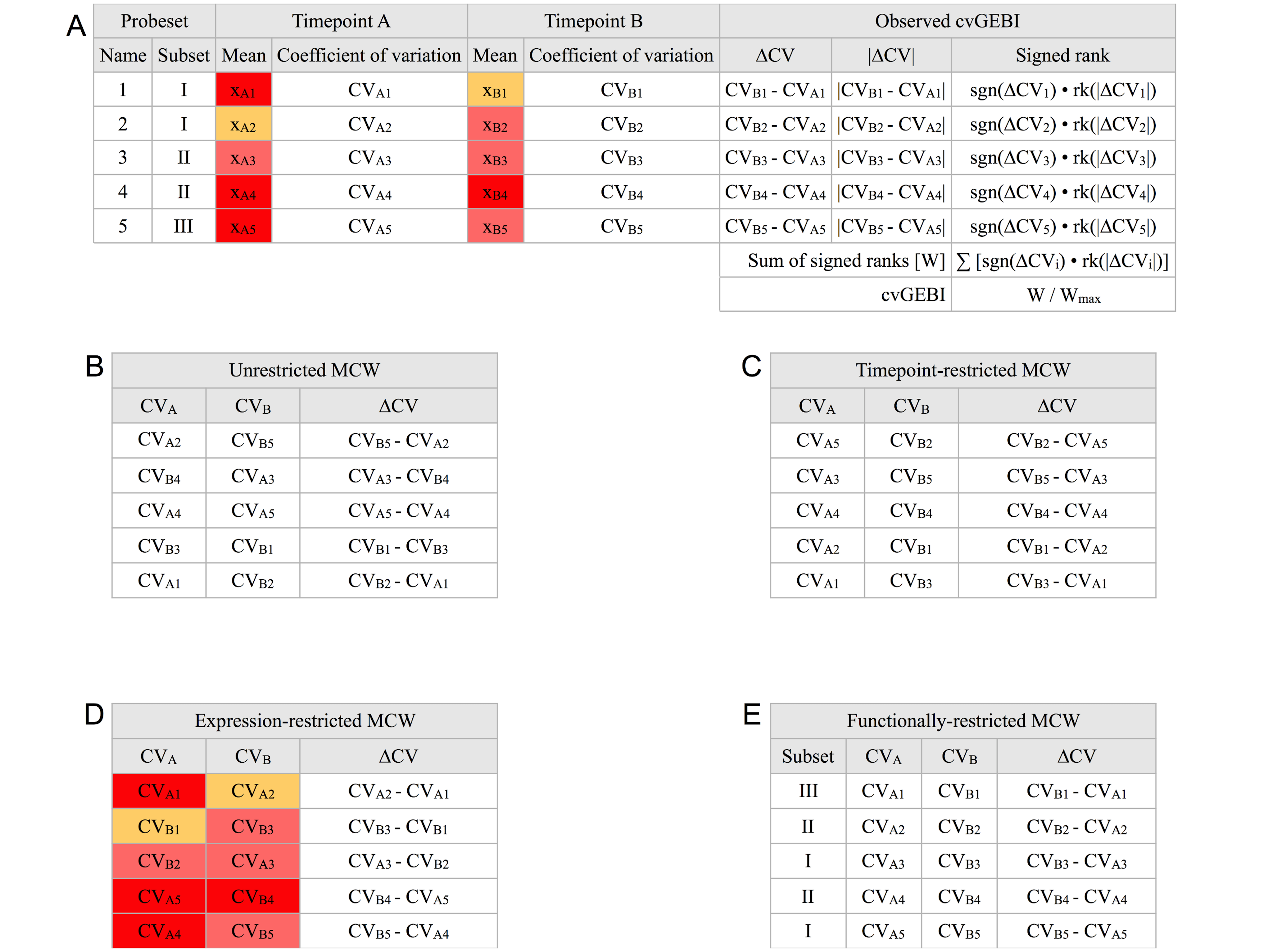

Supplement: Figure S2 — (A) Table representing transcript abundance mean and coefficient of variation (CV) for five transcripts and two timepoints, and the process followed to calculate gene expression bias index for transcript abundance CV (cvGEBI). Transcript abundance means were assorted in three bins represented with different colors according to their values. MCW tests evaluate the statistical significance of observed cvGEBIs by comparing them with cvGEBIs calculated after randomly rearranging transcript abundance CV. (B) Unrestricted MCW tests aims to simulate the effect of chance alone by calculating cvGEBIs after randomly rearranging transcript abundance CV with no restriction. (C) Timepoint-restricted MCW tests aim to simulate the effect factors acting on the transcriptome as a whole by calculating cvGEBIs after randomly rearranging transcript abundance CV respecting timepoint assortment. (D) Expression-restricted MCWs tests aim to simulate the effect of the mean level of expression by calculating cvGEBIs within bins defined by the absolute value of their corresponding transcript abundance mean. Colored boxes represent bins as defined in Table A. (E) Functionally-restricted MCWs tests aim to compare transcript abundance CV dynamics for subsets of genes defined by their functionality when compared with the whole transcriptome by calculating observed and simulated cvGEBIs before and after randomly rearranging functional subset tags. [file peerj-05-4004-s002.png]
