# Supplementary material for: Transcriptome dynamics along axolotl regenerative development are consistent with an extensive reduction in gene expression heterogeneity in dedifferentiated cells
Source: PeerJ. 2017 Nov 6;5:e4004. doi: 10.7717/peerj.4004 (PMC5678507; doi:10.7717/peerj.4004)
Supplement: Supplemental Information 1 — Díaz-Castillo (2017b). Theorizing about gene expression heterogeneity patterns after cell dedifferentiation and their potential value for regenerative engineering. In: Gardiner DM, ed. Regenerative Engineering and Developmental Biology: Principles and Applications. Florida: CRC Press, 351–360. [file peerj-05-4004-s009.pdf]

Chapter 15 Theorizing about gene expression heterogeneity patterns after cell dedifferentiation and their potential value for regenerative engineering

Carlos Díaz-Castillo

Contents

15.1 Introduction . . . 352
15.2 Cell dedifferentiation . . . 353
15.3 Cell dedifferentiation-based convergence . . . 354
15.4 Cell dedifferentiation-based reduction in gene expression noise . . . 355
15.5 Summary and perspectives . . . 356
Acknowledgments . . . 357
References . . . 357

> It is not just that noise is a bad signal. And it is not just that we want to actively reduce noise. We all want cleaner images and more reliable data lines and more soundproof walls and windows. We want more: We want to eliminate noise. We want to wipe noise out of digital existence. We want to win the war on noise through total annihilation.
>
> But we never will.
>
> **Bart Kosko**
> *(Kosko 2006)*

K25679_C015.indd 351 04/22/17 9:38:39 PM

> In short, variation is an endless source of challenging questions.
>
> **Ernst Mayr**
> *(Hallgrímsson and Hall 2011)*

Key concepts

a. Cell dedifferentiation, the process by which specialized cells are reverted to less specialized, more proliferative, and more pluripotent stages, is important for the regeneration of complex structures, both in plants and in animals.
b. Cell dedifferentiation might result in cell convergence; in other words, dedifferentiated cells would be more similar among themselves than the specialized cells they originated from were.
c. Information theory-based measures for transcriptome specialization and diversity from mouse and human tumors and the organ they originate from reflect the cell convergence that follows cell dedifferentiation.
d. Cell dedifferentiation commonly concurs with a general decompaction of chromatin across genomes.
e. Since chromatin compaction is an important enhancer of gene expression noise, it would be expected that gene expression became generally less noisy after cell dedifferentiation.
f. Transcriptome convergence and gene expression noise reduction after cell dedifferentiation should be manifested as a general reduction of gene expression heterogeneity, which might be very useful for the characterization of naturally occurring regenerative processes and the inception of regenerative engineering approaches.

15.1 Introduction

For ages, variation in living organisms has been a constant source of inspiration for biologists. However, it is humbling to acknowledge how little we now about the molecular and cellular bases of biological variation and its integration with deterministic mechanisms to explain life or to find applied solutions to human troubles (Hallgrímsson and Hall 2011). Gene expression, the concatenation of steps toward the production of active RNAs and proteins from instructions encoded in the genome, is particularly suitable to study the molecular and cellular bases of biological variation. Gene expression can vary due to genetic changes in elements that participate in its regulation, in response to environmental changes and/or developmental processes, and also in the absence of any of these inputs (Kaern et al. 2005, Raser and O'Shea 2005, Raj and van Oudenaarden 2008, Kilfoil et al. 2009). In addition, gene expression variation results

in a phenotypic variation that can be manifested as heterogeneity at different levels, from cells within a tissue to individuals within a population, with an important effect on normal and pathological development or the evolution of species (Kaern et al. 2005, Raser and O'Shea 2005, Raj and van Oudenaarden 2008, Kilfoil et al. 2009). Thus, the study of gene expression variation might permit disentangling the factors contributing to biological variation and how these factors might influence the course of developmental, pathological, or evolutionary processes.

Regenerative engineering represents a brave attempt of scientific and technological convergence, with concrete goals toward the regeneration of complex structures (Laurencin and Nair 2015). The completion of regeneration of human joints and whole limbs has been scheduled for 2022 and 2030, respectively (Khademhosseini 2015), forcing us to explore even the most unlikely avenue of potential convergence toward these goals. Traditionally, some of the main areas contributing to regenerative engineering have been heavily invested toward the study of conserved principles—developmental biology—or even dismissive of the utility of manifestations of variation such as noise—engineering. In the present essay, I reflect on two particular aspects associated with cell dedifferentiation to suggest that gene expression heterogeneity across genomes will decrease in events that course with cell dedifferentiation: plant somatic embryogenesis, cancer, or regenerative development. I kept this piece simple and concise because it is considerably speculative and it is aimed to elicit a biological variation-friendly mindset in regenerative engineers, while pointing to basic researchers the utility that the study of regenerative development might have for the understanding of the molecular and cellular bases of biological variation. A manuscript reporting preliminary analyses that confirm the central hypothesis drawn here is currently in preparation and will be published separately.

15.2 Cell dedifferentiation

From a cellular point of view, the development of multicellular organisms is a process characterized by cell differentiation: the progressive and coordinated specialization and loss of proliferative potential of initially pluripotent cells. The study of cell differentiation is one of the main pillars of developmental biology. Far less studied, cell dedifferentiation, or the cases in which specialized cells are reverted to less specialized, more proliferative, and more pluripotent stages, is important for the formation of naturally occurring calli and tumors or the regeneration of complex structures in both plants and animals (King and Newmark 2012, Ikeuchi et al. 2013, Friedmann-Morvinski and Verma 2014, Varga et al. 2014, Yamada et al. 2014, Campos-Sanchez and Cobaleda 2015, Grafi and Barak 2015, Li et al. 2015, Sugiyama 2015). In addition, cell dedifferentiation is important for induced somatic embryogenesis in plants or human pluripotent stem cells (induced pluripotent stem cells [iPSCs]) AQ 2

(Jopling et al. 2011, Kami and Gojo 2014, El-Badawy and El-Badri 2015, Feher 2015, Krause et al. 2015, Sugiyama 2015). Thus, the understanding of dedifferentiation dynamics is of broad interest for plant biotechnology or medicine and especially for regenerative engineering.

Two features that are characteristic of dedifferentiating cells permit speculating that a general reduction in gene expression heterogeneity might be observable for events that course with cell dedifferentiation. These two features are the cellular convergence that cell dedifferentiation represents and the general reduction in chromatin compaction characteristic of cell dedifferentiation that might cause a reduction in gene expression noise.

15.3 Cell dedifferentiation-based convergence

Since all specialized cells within an organism ultimately originate from a single cell, the zygote, the mere idea that specialized cells can be reverted to previous pluripotent stages implies that events that course with cell dedifferentiation could be conceptualized as cases of cell convergence; *in other words*, dedifferentiated cells would be more similar among themselves than the specialized cells they originated from are. Whether all cell dedifferentiations reach the same level of undifferentiated pluripotency is still contentious. For example, iPSCs and tumor cells are supposed to be reverted to a more ancestral level of pluripotency than dedifferentiated cells in salamander limb regeneration, which seem to maintain some memory of the differentiated lineage from which they come (Kragl et al. 2009, Eguizabal et al. 2013, Yamada et al. 2014, Campos-Sanchez and Cobaleda 2015). Notwithstanding these differences, if cell dedifferentiation truly embodied a convergence of cells toward undifferentiated stages, this convergence could be detected as a reduction in heterogeneity for cellular features.

Interestingly, the putative convergence of dedifferentiated cells might be detectable at the transcriptomic level. In two different articles, Martínez el al. used information theory to derive two measures for specialization and diversity of transcriptomes from different tissues and applied them to the study of transcriptomes obtained from different human and murine cancers and the tissues from which they originated (Martinez and Reyes-Valdes 2008, Martinez et al. 2010). These authors showed that despite their different independent origins, the transcriptomes of most cancers show a very similar reduction in the level of expression for tissue-specific genes and a homogenization of the level of expression for expressed genes when compared with the tissues from which they originated (Martinez et al. 2010). Furthermore, cancer transcriptomes seem to be very similar in terms of specialization and divergence to the transcriptome of undifferentiated embryonic stem cells, underscoring the potential convergence putatively associated with cell dedifferentiation (Martinez et al. 2010). Whether similar trends are visible in all events AQ 3 coursing with cell dedifferentiation is still incognito.

15.4 Cell dedifferentiation-based reduction in gene expression noise

Recently, the study of gene expression in populations of clonal cells maintained in the same environment has suggested that gene expression is prone to vary even in the absence of genetic, environmental, or developmental cues (Kaern et al. 2005, Raser and O'Shea 2005, Raj and van Oudenaarden 2008, Kilfoil et al. 2009). Like many other intracellular processes, gene expression relies on a few copies of many intervening elements acting amidst very congested intracellular contexts, and therefore, it is susceptible to stochastic fluctuations of both required and contextual molecules (Kaern et al. 2005, Raser and O'Shea 2005, Raj and van Oudenaarden 2008, Kilfoil et al. 2009). The variation in gene expression that ultimately depends on molecular stochasticity is commonly referred to as stochastic variation in gene expression, or gene expression noise. The characterization of gene expression noise is still in its infancy, and many aspects are yet to be clarified. For example, it is unclear how many cells within a population could be considered in comparable microenvironments and developmental/cell cycle states, so the variation they show can be unambiguously deemed as stochastic or if stochastic variation caused at any of the steps of gene expression is mitigated in subsequent steps or propagated through biological systems, resulting in phenotypic noise (Battich et al. 2015).

One interesting aspect for gene expression noise is that it might be variable itself, being such a variation that is potentially very informative about nuclear dynamics of cells involved in certain processes. One of the factors that contribute to the variation in gene expression noise is chromatin compaction (Kaern et al. 2005, Raser and O'Shea 2005, Raj and van Oudenaarden 2008). Slow dynamics for the transition between compacted and open chromatin states for regions with highly compacted chromatin or heterochromatin make the expression of genes located there particularly noisy (Kaern et al. 2005, Raser and O'Shea 2005, Raj and van Oudenaarden 2008). Although the study of gene expression noise in clonal cells is recent (Kaern et al. 2005, Raser and O'Shea 2005, Raj and van Oudenaarden 2008), an extensive literature exist on stochastic patterns of gene expression variation for genes located close to or within heterochromatin (reviewed in Elgin and Reuter 2013). Position-effect variegation (PEV) was first discover in *Drosophila,* associated with the relocation of a gene close to heterochromatin, caused by a chromosome inversion, and since then, it has been extended to other species for genes located close to or within heterochromatin (Elgin and Reuter 2013). The expression of these genes show signs of stochastic variation with regard to their state of activation/repression, their level of expression for cells of the same individual, and their expression patterning between individuals of the same progeny (Elgin and Reuter 2013). Since chromatin compaction for many genes can change during the cell cycle, between cell types, or in response to biotic and abiotic signals, it would

K25679_C015.indd 355 04/22/17 9:38:39 PM

be expected that chromatin compaction–dependent gene expression noise across genomes was also variable. In fact, it is known that factors that affect chromatin compaction, such as temperature, genetic variation in heterochromatin-forming elements, and the direction of chromosome inheritance, result in the modification of stochastic patterns in gene expression, such as PEV (Maggert and Golic 2002, Elgin and Reuter 2013). Furthermore, recently, it has been suggested that large differences in junk DNA genomic content between individuals of different sexes in the same species can cause a variation in gene expression noise, with an important effect on the expression of phenotypes and species dynamics (Diaz-Castillo 2015).

Interestingly, both naturally occurring and induced dedifferentiation events are characterized by a general opening of the chromatin (Jiang et al. 2013, El-Badawy and El-Badri 2015, Feher 2015, Grafi and Barak 2015, Jiang et al. 2015, Krause et al. 2015, Lee et al. 2015). Such changes would result in many genes to become more accessible to the basic AQ 4 machinery and regulatory elements of transcription. In fact, the early expression of otherwise-silent transposable elements in dedifferentiating cells is consistent with the derived accessibility of many loci in the genome that are associated with the general opening of the chromatin (Wang and Wang 2012, Zhu et al. 2012, Macia et al. 2015). Considering that chromatin compaction is an important enhancer for gene expression noise (Kaern et al. 2005, Raj and van Oudenaarden 2008, Raser and O'Shea 2005), it could be inferred that the extensive reduction in chromatin compaction across dedifferentiating nuclei would cause a general reduction in gene expression noise. Whether events that course with cell dedifferentiation are truly characterized by a general decrease in gene AQ 5 expression noise is also incognito.

15.5 Summary and perspectives

In this brief essay, I hypothesized that processes that concur with cell dedifferentiation would do so with a general decrease in gene expression heterogeneity because of the convergence that cell dedifferentiation represents itself and the general reduction in gene expression noise caused by the generalized opening of the chromatin, characteristic AQ 6 of dedifferentiating cells. To the best of my knowledge, no literature exists that directly addresses gene expression heterogeneity dynamics along processes that concur with cell dedifferentiation. Limb regeneration in salamanders might be a very suitable model system to test the validity of this hypothesis and explore its value further for regenerative engineering. In recent years, a number of studies analyzed transcriptome dynamics along limb regeneration in *Ambystoma mexicanum* or Mexican axolotl (Monaghan et al. 2009, Monaghan et al. 2012, Stewart et al. 2013, Wu et al. 2013, McCusker et al. 2015, Voss et al. 2015). Although most of these studies have low levels of biological replication,

K25679_C015.indd 356 04/22/17 9:38:39 PM

which would be an inconvenience for the study of gene expression heterogeneity, they can be used to make preliminary observations and better design-specific studies in the future. Moreover, despite the difficulty that entails sequencing large genomes cluttered with large amounts of repetitive DNA, the genome of the axolotl is currently being sequenced, and soon, it would be feasible to relate gene expression heterogeneity dynamics with other aspects of the nuclear architecture (Keinath et al. 2015). Although it is still too soon to appreciate the value of the putative reduction in gene expression heterogeneity along processes that concur with cell dedifferentiation for regenerative engineering, the study of gene expression heterogeneity along limb regeneration might help to better characterize gene expression dynamics along processes where cells dedifferentiate, to identify elements that might be important for the regulation of these processes, or to locate chromosome domains with particular gene expression heterogeneity dynamics that can be used as target for the insertion of reporter genes to help better monitor the correct progress of regenerative processes by using non-invasive methodologies.

Acknowledgments

I want to express my deepest gratitude to Raquel Chamorro-García for her unfailing support and to David Gardiner for thinking that there is room within the regenerative engineering arena for thoughts on biological variation.

References

Battich, N., T. Stoeger, and L. Pelkmans. 2015. Control of transcript variability in single mammalian cells. *Cell* 163 (7):1596–1610. doi:10.1016/j.cell.2015.11.018.

Campos-Sanchez, E. and C. Cobaleda. 2015. Tumoral reprogramming: Plasticity takes a walk on the wild side. *Biochim Biophys Acta* 1849 (4):436–447. doi:10.1016/j.bbagrm.2014.07.003.

Diaz-Castillo, C. 2015. Evidence for a sexual dimorphism in gene expression noise in metazoan species. *PeerJ* 3:e750. doi:10.7717/peerj.750.

***This article shows that in metazoans, gene expression noise might be generally larger for heterogametic individuals than for homogametic individuals and that such sexual dimorphism might be dependent on chromatin formation early in embryogenesis, in the presence or absence of junk DNA–enriched sex-specific chromosomes.

Eguizabal, C., N. Montserrat, A. Veiga, and J. C. I. Belmonte. 2013. Dedifferentiation, transdifferentiation, and reprogramming: Future directions in regenerative medicine. *Semin Reprod Med* 31 (1):82–94. doi:10.1055/s-0032-1331802.

El-Badawy, A. and N. El-Badri. 2015. Regulators of pluripotency and their implications in regenerative medicine. *Stem Cells Cloning* 8:67–80. doi:10.2147/SCCAA.S80157.

Elgin, S. C. and G. Reuter. 2013. Position-effect variegation, heterochromatin formation, and gene silencing in Drosophila. *Cold Spring Harb Perspect Biol* 5 (8):a017780. doi:10.1101/cshperspect.a017780.

***This review is a great segue into the extensive literature on the phenomenon referred to as position-effect variegation (PEV)—the stochastic phenotypic variation derived from genes being located close to or within chromosome regions with highly compacted chromatin or heterochromatin.

Feher, A. 2015. Somatic embryogenesis - Stress-induced remodeling of plant cell fate. *Biochim Biophys Acta* 1849 (4):385–402. doi:10.1016/j.bbagrm.2014.07.005.

Friedmann-Morvinski, D. and I. M. Verma. 2014. Dedifferentiation and reprogramming: Origins of cancer stem cells. *EMBO Rep* 15 (3):244–253. doi:10.1002/embr.201338254.

Grafi, G. and S. Barak. 2015. Stress induces cell dedifferentiation in plants. *Biochim Biophys Acta* 1849 (4):378–384. doi:10.1016/j.bbagrm.2014.07.015.

Hallgrímsson, B. and B. K. Hall. 2011. *Variation: A Central Concept in Biology*. Academic Press: New York.

Ikeuchi, M., K. Sugimoto, and A. Iwase. 2013. Plant callus: Mechanisms of induction and repression. *Plant Cell* 25 (9):3159–3173. doi:10.1105/tpc.113.116053.

Jiang, F., Z. Feng, H. Liu, and J. Zhu. 2015. Involvement of plant stem cells or stem cell-like cells in dedifferentiation. *Front Plant Sci* 6:1028. doi:10.3389/fpls.2015.01028.

Jiang, F., J. Zhu, and H. L. Liu. 2013. Protoplasts: A useful research system for plant cell biology, especially dedifferentiation. *Protoplasma* 250 (6):1231–1238. doi:10.1007/s00709-013-0513-z.

Jopling, C., S. Boue, and J. C. I. Belmonte. 2011. Dedifferentiation, transdifferentiation and reprogramming: Three routes to regeneration. *Nat Rev Mol Cell Biol* 12 (2):79–89. doi:10.1038/nrm3043.

Kaern, M., T. C. Elston, W. J. Blake, and J. J. Collins. 2005. Stochasticity in gene expression: From theories to phenotypes. *Nat Rev Genet* 6 (6):451–464. doi:10.1038/nrg1615.

***This is a great review on stochastic biological variation—the variation observed in biological systems, even in the absence of genetic or environmental cues.

Kami, D. and S. Gojo. 2014. Tuning cell fate: From insights to vertebrate regeneration. *Organogenesis* 10 (2):231–240. doi:10.4161/org.28816.

Keinath, M. C., V. A. Timoshevskiy, N. Y. Timoshevskaya, P. A. Tsonis, S. R. Voss, and J. J. Smith. 2015. Initial characterization of the large genome of the salamander Ambystoma mexicanum using shotgun and laser capture chromosome sequencing. *Sci Rep* 5:16413. doi:10.1038/srep16413.

Khademhosseini, A. 2015. HEAL project aims to regenerate human limbs by 2030. *Regen Eng Transl Med* 1 (1–4):50–57. doi:10.1007/s40883-015-0007-y.

Kilfoil, M. L., P. Lasko, and E. Abouheif. 2009. Stochastic variation: From single cells to superorganisms. *HFSP J* 3 (6):379–385. doi:10.2976/1.3223356.

***This is a great review on stochastic biological variation—the variation observed in biological systems, even in the absence of genetic or environmental cues.

King, R. S. and P. A. Newmark. 2012. The cell biology of regeneration. *J Cell Biol* 196 (5):553–562. doi:10.1083/jcb.201105099.

Kosko, B. 2006. *Noise*. Viking: New York.

Kragl, M., D. Knapp, E. Nacu, S. Khattak, M. Maden, H. H. Epperlein, and E. M. Tanaka. 2009. Cells keep a memory of their tissue origin during axolotl limb regeneration. *Nature* 460 (7251):60–65. doi:10.1038/nature08152.

Krause, M. N., I. Sancho-Martinez, and J. C. I. Belmonte. 2015. Understanding the molecular mechanisms of reprogramming. *Biochem Biophys Res Commun* 473 (3): 693–697. doi:10.1016/j.bbrc.2015.11.120.

Laurencin, C. T. and L. S. Nair. 2015. Regenerative engineering: Approaches to limb regeneration and other grand challenges. *Regen Eng Transl Med* 1 (1–4):1–3. doi:10.1007/s40883-015-0006-z.

Lee, K., O. S. Park, S. J. Jung, and P. J. Seo. 2015. Histone deacetylation-mediated cellular dedifferentiation in Arabidopsis. *J Plant Physiol* 191:95–100. doi:10.1016/j.jplph.2015.12.006.

K25679_C015.indd 358 04/22/17 9:38:39 PM

Li, Q., H. Yang, and T. P. Zhong. 2015. Regeneration across metazoan phylogeny: Lessons from model organisms. *J Genet Genomics* 42 (2):57–70. doi:10.1016/j.jgg.2014.12.002.

Macia, A., E. Blanco-Jimenez, and J. L. Garcia-Perez. 2015. Retrotransposons in pluripotent cells: Impact and new roles in cellular plasticity. *Biochim Biophys Acta* 1849 (4):417–426. doi:10.1016/j.bbagrm.2014.07.007.

Maggert, K. A. and K. G. Golic. 2002. The Y chromosome of Drosophila melanogaster exhibits chromosome-wide imprinting. *Genetics* 162 (3):1245–1258.

Martinez, O. and M. H. Reyes-Valdes. 2008. Defining diversity, specialization, and gene specificity in transcriptomes through information theory. *Proc Natl Acad Sci USA* 105 (28):9709–9714. doi:10.1073/pnas.0803479105.

Martinez, O., M. H. Reyes-Valdes, and L. Herrera-Estrella. 2010. Cancer reduces transcriptome specialization. *PLoS One* 5 (5):e10398. doi:10.1371/journal.pone.0010398.

***This and the previous reference use information theory to define measures for transcriptome specialization and divergence and apply them to the study of transcriptomes from mouse and human tumors and the organs from which they originated. Their results are consistent with the hypothesized cellular convergence associated with cell dedifferentiation.

McCusker, C. D., A. Athippozhy, C. Diaz-Castillo, C. Fowlkes, D. M. Gardiner, and S. R. Voss. 2015. Positional plasticity in regenerating Amybstoma mexicanum limbs is associated with cell proliferation and pathways of cellular differentiation. *BMC Dev Biol* 1–17. doi:10.1186/s12861-015-0095-4.

Monaghan, J. R., A. Athippozhy, A. W. Seifert, S. Putta, A. J. Stromberg, M. Maden, D. M. Gardiner, and S. R. Voss. 2012. Gene expression patterns specific to the regenerating limb of the Mexican axolotl. *Biol Open* 1 (10):937–948. doi:10.1242/bio.20121594.

Monaghan, J R., L. G. Epp, S. Putta, R. B. Page, J. A. Walker, C. K. Beachy, W. Zhu et al. 2009. Microarray and cDNA sequence analysis of transcription during nerve-dependent limb regeneration. *BMC Biol* 7 (1):1. doi:10.1186/1741-7007-7-1.

Raj, A. and A. van Oudenaarden. 2008. Nature, nurture, or chance: Stochastic gene expression and its consequences. *Cell* 135 (2):216–226. doi:10.1016/j.cell.2008.09.050.

***This is a great review on stochastic biological variation—the variation observed in biological systems, even in the absence of genetic or environmental cues.

Raser, J. M. and E. K. O'Shea. 2005. Noise in gene expression: Origins, consequences, and control. *Science* 309 (5743):2010–2013. doi:10.1126/science.1105891.

***This is a great review on stochastic biological variation—the variation observed in biological systems, even in the absence of genetic or environmental cues.

Stewart, R., C. A. Rascón, S. Tian, J. Nie, C. Barry, L.-F. Chu, H. Ardalani et al. 2013. Comparative RNA-seq analysis in the unsequenced axolotl: The oncogene burst highlights early gene expression in the blastema. *PLoS Comp Biol* 9 (3):e1002936. doi:10.1371/journal.pcbi.1002936.

Sugiyama, M. 2015. Historical review of research on plant cell dedifferentiation. *J Plant Res* 128 (3):349–359. doi:10.1007/s10265-015-0706-y.

Varga, J., T. D. Oliveira, and F. R. Greten. 2014. The architect who never sleeps: Tumor-induced plasticity. *FEBS Lett* 588 (15):2422–2427. doi:10.1016/j.febslet.2014.06.019.

Voss, S. R., A. Palumbo, R. Nagarajan, D. M. Gardiner, K. Muneoka, A. J. Stromberg, and A. T. Athippozhy. 2015. Gene expression during the first 28 days of axolotl limb regeneration I: Experimental design and global analysis of gene expression. *Regeneration* 2 (3):120–136. doi:10.1002/reg2.37.

K25679_C015.indd 359 04/22/17 9:38:39 PM

Wang, Q. M. and L. Wang. 2012. An evolutionary view of plant tissue culture: Somaclonal variation and selection. *Plant Cell Rep* 31 (9):1535–1547. doi:10.1007/s00299-012-1281-5.

Wu, C-H., M.-H. Tsai, C.-C. Ho, C.-Y. Chen, and H.-S. Lee. 2013. De novo transcriptome sequencing of axolotl blastema for identification of differentially expressed genes during limb regeneration. *BMC Genomics* 14 (1):1. doi:10.1186/1471-2164-14-434.

Yamada, Y., H. Haga, and Y. Yamada. 2014. Concise review: Dedifferentiation meets cancer development: Proof of concept for epigenetic cancer. *Stem Cells Transl Med* 3 (10):1182–1187. doi:10.5966/sctm.2014-0090.

Zhu, W., D. Kuo, J. Nathanson, A. Satoh, G. M. Pao, G. W. Yeo, S. V. Bryant, S. R. Voss, D. M. Gardiner, and T. Hunter. 2012. Retrotransposon long interspersed nucleotide element-1 (LINE-1) is activated during salamander limb regeneration. *Dev Growth Differ* 54 (7):673–685. doi:10.1111/j.1440-169X.2012.01368.x.

K25679_C015.indd 360 04/22/17 9:38:39 PM
